# The Potential of Purinergic Signaling to Thwart Viruses Including SARS-CoV-2

**DOI:** 10.3389/fimmu.2022.904419

**Published:** 2022-06-17

**Authors:** Davide Ferrari, Michele Rubini, Jorge S. Burns

**Affiliations:** ^1^ Section of Microbiology and Applied Pathology, University of Ferrara, Ferrara, Italy; ^2^ Department of Life Science and Biotechnology, University of Ferrara, Ferrara, Italy; ^3^ Department of Neuroscience and Rehabilitation, University of Ferrara, Ferrara, Italy; ^4^ Department of Environmental and Prevention Sciences, University of Ferrara, Ferrara, Italy

**Keywords:** extracellular ATP, extracellular adenosine, P1 receptors, P2 receptors, virus, SARS-CoV-2

## Abstract

A long-shared evolutionary history is congruent with the multiple roles played by purinergic signaling in viral infection, replication and host responses that can assist or hinder viral functions. An overview of the involvement of purinergic signaling among a range of viruses is compared and contrasted with what is currently understood for SARS-CoV-2. In particular, we focus on the inflammatory and antiviral responses of infected cells mediated by purinergic receptor activation. Although there is considerable variation in a patient’s response to SARS-CoV-2 infection, a principle immediate concern in Coronavirus disease (COVID-19) is the possibility of an aberrant inflammatory activation causing diffuse lung oedema and respiratory failure. We discuss the most promising potential interventions modulating purinergic signaling that may attenuate the more serious repercussions of SARS-CoV-2 infection and aspects of their implementation.

## Introduction to the Purinome and its Druggability

Of broad function, purine containing nucleotides and nucleosides serve as the precursors of RNA and DNA and also have ubiquitous critical roles as cofactors in the enzymatic reactions that create and sustain all living organisms. These include small G proteins, kinases, dehydrogenases, ATPases, non-conventional purine utilizing enzymes, helicases, synthetases, deaminases, lipases, sulfotransferases, cytosolic tyrosine kinases, carboxylases, motor proteins and purinergic receptors. Adenosine 5’-Triphosphate (ATP) of high abundance in every cell (1-10 mM) represents an archetypical molecule that intracellularly is a fundamental energy currency, providing controlled bursts of about 7 kilocalories per mole with the splitting of its terminal phosphoanhydride bond yielding adenosine 5’-diphosphate (ADP) and inorganic phosphate (Pi). The proposition that extracellularly, the ATP molecule also served as a signaling cotransmitter, was confirmed with the cloning and characterization of specific receptors for purines and pyrimidines (1). The large family of receptors has been subdivided into three subtypes according to molecular, biochemical and pharmacological characteristics; comprising four members of the P1 (adenosine) receptor family, seven members of the P2X receptor family and eight members of the P2Y receptor family. These receptors interact with a vast heterogeneity of purinergic ligands that in turn are regulated in substance and concentration by ectonucleotide-metabolizing enzymes hydrolyzing nucleotide phosphates and the activity of nucleotide channels and transporters. The extracellular purine signaling network constitutes only a small percentage of the more than 3,260 proteins utilizing purine cofactors that constitute the total human purinome (2). However, since inter-cellular communication represents a powerful functional class comprised of significant complex and dynamic molecular cross-talk networks, this has itself been termed “purinome”, comprising the numerous extracellular molecular interaction partners responsible for the biological effects of extracellular purine and pyrimidine ligands (3). This review will refer to the total purinome when considering virus interactions with intracellular host proteins that may dictate viral replication, yet will predominantly focus on the extracellular definition of “purinome” and the associated concept of “receptosome” comprising the web of interactions between not only receptors but other proteins at the plasma membrane that contribute to the very heterogeneous responses to epigenetic factors and modified extracellular environments. The receptosome presents a comprehensive regulatory concept of dynamic interactions that collectively invoke the fine tuning to accommodate rapidly changing microenvironmental contexts (4). The receptosome concept is thus broader than the similar-sounding yet alternatively defined “recepterome” of plasma membrane hosted ligand capture molecules that serve as a useful cohort to efficiently discover molecular targets for therapeutic drug discovery (5).

Capture of purinome molecular participants has been achieved using affinity arrays of γ-phosphate linked ATP-sepharose, orientated towards ligand-relevant conformations, to screen small molecule libraries (6, 7). This has generated information about drug targets and also off-target liabilities, an important consideration given that shared molecular spaces within purine binding pockets are not likely to be very diverse, with high probability that seemingly disparate enzymes may bind the same drug by chance. Several hundred purine-utilizing enzymes are implicated in the Online Mendelian Inheritance in Man (OMIM) database as being associated with inborn errors of metabolism, making the unwanted off-target side-effects more likely. Nonetheless, the advantageous ability to simultaneously screen for potency and selectivity has meant drugs targeting purine-utilizing enzymes have been very successful. Galdanamycin that binds the ATP/ADP binding pocket in the N terminus of the HSP90 protein has been shown to inhibit proliferation of a wide variety of tumor cell lines and possess antifungal activity (8).

The anti-folate agent Methotrexate (MTX), a folic acid analogue that is polyglutamated inside the cell to its active form, can inhibit *de novo* purine synthesis involved in generating the precursor nucleotides required for RNA and DNA. Besides being a chemotherapy agent and immune system suppressant, MTX has also some antiviral effect, as it can reduce the viral titer of Zika virus, which can be rescued through addition of adenosine to virus-infected cells. This evidence suggests that restriction of *de novo* synthesis ATP pools could suppress viral replication (9). In addition, this disrupts the S phase of the cell cycle in rapidly dividing immune cells and additional immunomodulatory effects mean that MTX can reduce inflammation and joint damage in some rheumatological diseases. However, acute high dose methotrexate treatment for cancer is associated with lactic acidosis and even when used at low doses for arthritis, there is a risk of liver fibrosis (10). Thus when developing pharmacological approaches that target antiviral purinergic mechanisms there is need to go beyond single molecule concepts and adopt a more holistic approach. New public databases of AI-predicted protein structures, incorporating AlphaFold or RoseTTAFold algorithms (11, 12), to improve computational prediction accuracy of protein conformations, could be invaluable for accelerating the development of purinome directed drugs.

## Virus-Cell Purinome Interactions Share Ancient History

Contemporary to early stages of the evolution of life, chemically treated and irradiated powdered meteorites are demonstrable sources of amino acids and nucleosides (13). It is considered that within a primordial context emerged viroids, virus-like infectious agents; fundamentally an encapsulated biological code, acquiring biotic potency once the code could be processed and directed towards self-assembly of a unit particle (14). To what extent viruses are likely to have evolved from viroid-like RNA that preceded cellular life (15) is of debate (16). However, the relatively recent discovery of giant viruses, that can mimic bacteria in size, with >1000 gene genomes including genes more significant for cellular rather than viral metabolism, encoding a large yet incomplete repertoir of protein-synthesis translation factors (17), has lent credence to the concept that early plant-interacting viroids were autonomous units.

Selection pressure for increased autonomy has resulted in self-replicating cells and specialisations coordinated to the level of whole organisms that derive nutrition and energy from environmental interactions that sustain life. Molecules serving roles restricted to replication have been adopted for more complex tasks governing intracellular and ultimately intercellular signaling. Conversely, evolutionary pressures favouring selection for replication with reduced energy requirements, exploited endosymbiotic interactions to such a parasitic extent that today’s viruses lack enough genes to live independently.

Given a replicative code chemistry composed of ribonucleic acid (RNA) or deoxyribonucleic acid (DNA), viruses have a critical requirement for purines and pyrimidines as essential small molecules constituting the bases of synthesized nucleotides. Within a cellular microenvironment, these can be sourced from *de novo* pathways of synthesis or obtained from more energetically efficient salvage pathways whereby component small molecules, such as hypoxanthine, adenine or guanine for purine synthesis or uracil, cytidine or thymidine for pyrimidine are synthesized from catabolic products of nucleic acid turnover, processes that may be therapeutically exploited (18). Characterization of specific cellular receptors for extracellular nucleotides has revealed a repurposing of purine nucleotides and nucleosides. They can also act as extracellular signaling mediators, as ligands that interact with a broad array of purinoreceptors and ectonucleotidases in different tissues and infiltrating immune cells; thereby governing diverse functions in almost every organ and system of organisms. The long shared history of viruses and the human purinome, provides ancient interplay for evolving intricate and complex interactions pertaining to both viral establishment within host cell metabolism, cell-reactive mechanisms aiming to restore homeostasis and viral countermeasures to host defense mechanisms to maintain selfish genetic presence.

Five steps of viral infection include; (i) Binding to a host cell, (ii) receptor downstream interactions facilitating entry and integration within the host cell genome, (iii) Genetic code processing to favor viral genome replication, (iv) Processing of the viral code to commandeer host cell functions supporting translation and assembly of viral core structures, (v) Release of maturated viral particles into the microenvironment allowing recapitulation of processes (i) to (v) in new host cells. It is not surprising that purinergic signaling plays a significant role in all these viral infection steps and host cell responses (19), the challenge is to characterize precisely how viral interactions can impact upon purinergic signaling to influence pathological processes and whether this can reveal key targets for effective therapeutic intervention.

By reviewing responses to viral infection mediated by purinergic signaling discovered in a diverse array of antecedent viruses, we seek to establish clues and principles that may improve our understanding for better control of the newly emerged SARS-CoV-2 pandemic virus (20) and its COVID-19 disease symptoms.

## Extracellular Nucleotides and Nucleosides Act as Signaling Molecules

Undoubtedly, nucleotides (ATP, ADP, Uridine triphosphate (UTP), Uridine diphosphate (UDP), UDP- glucose) and nucleosides (adenosine/ADO) are fundamental for cell energy demands, metabolic regulation and building macromolecules such as nucleic acids (21). However, an increased opportunity to control viral pathologies is presented by focus on the additional purinergic role outside the cell, were they behave as intercellular ligands for signals acting both autocrinally and paracrinally to stimulate a plethora of organ and cell responses.

Besides being released as a consequence of membrane stress or damage, ATP can be transferred into the extracellular space by Pannexin-1, Connexin 43, exosomes and microvesicles (22). Resulting responses range from nervous transmission (23, 24), modulation of the cardiovascular system (25), muscle contraction (26), microvessel permeability (27), immune defense (28) and the fundamental processes of cell proliferation, differentiation and cell death (29–31).

**Figure 1:**
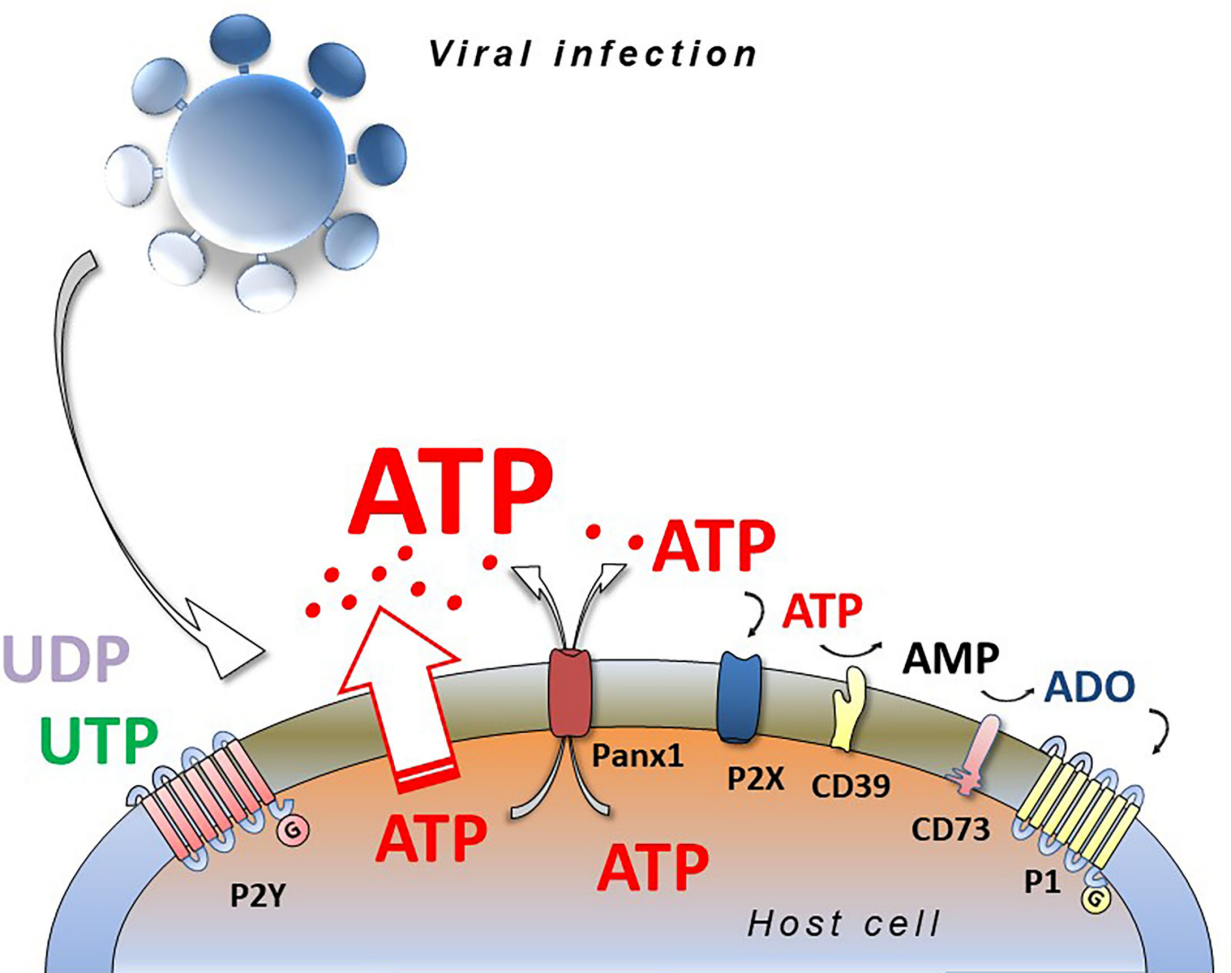
Viral infection and purinergic signalling. Viruses infecting eucariotic cells induce the release of intracellular nucleotides (ATP, ADP, UTP, UDP) by different ways (connexins, pannexins, membrane stress or damage) activating purinergic P2 (P2X and P2Y) and P1 (A_1_, A_2A_, A_2B_, A_3_) receptors and inducing pro- or anti-viral responses depending on virus species, cell type and purinergic receptors expressed. ATP and ADP are hydrolyzed by CD39 and CD73 ecto-nucleotidases generating adenosine (ADO) that is agonist at P1 receptors.

Responses to extracellular nucleotides and nucleosides occur by stimulation of plasma membrane proteins named purinergic P1 and P2 receptors. Extracellular ADO binds and activates P1 receptors that are G-protein-coupled, metabotropic seven-membrane-spanning domain receptors, subdivided into A_1_, A_2A_, A_2B_ and A_3_ receptors (32). The nomenclature for the diverse family of P2 receptors has bifurcated according to biochemical characteristics; ionotropic P2X and metabotropic P2Y forms. ATP, ADP, UTP, UDP, UDP-glucose activate P2 receptors, comprising seven P2X subtypes (P2X1-7) and eight G-protein-coupled P2Y receptor subtypes (P2Y_1, 2, 4, 6, 11, 12, 13, 14_) (33). The receptors have distinct preferential binding avidities for different extracellular nucleotides and ectonucleotidases, present on the extracellular side of the plasma membrane, that play a fundamental role in hydrolyzing nucleotides, thereby modifying their extracellular concentration and availability for the P1 and P2 receptors (34) (**Figure 1**).

## Release of Intracellular Nucleotides: A Widespread Paradigm for Cell Invading Microorganisms, Including Viruses

Disruption of cellular metabolic processes by “foreign agents” such as microbes and parasites e.g. *Plasmodium falciparum* infecting human erythrocytes (35), often evokes nucleotide release from infected cells, with extracellular ATP an exemplary contributor to danger-associated molecular patterns (DAMPs) (36). The DAMP response can include neutrophil recruitment (37), macrophage activation (38) and systemic inflammation (39), cellular pathways favoring elimination of different intracellular parasites (28, 40) and this likely explains why several microorganisms express ectonucleotidases that rapidly degrade released ATP to mitigate ATP-mediated cell lysis (41). Cytomegalovirus (CMV) infection induces adenine nucleotide liberation from endothelial cells (42) with disruption of ATP- and KCL-stimulated calcium signaling (43). Even singular viral components were able to provoke this response, although it is to be appreciated that sterile non-infectious trauma can also activate DAMPs and cause a systemic inflammatory response syndrome (39). Liberation of these nucleotides has been detected in numerous examples of virus infected cells including Herpes Simplex Virus 1 (HSV-1), Newcastle disease virus and murine leukemia virus (44). The HIV-1 glycoprotein gp120 induced ATP release from human macrophages and the nucleotide was also found in supernatants of HIV-1 infected cells (19, 45, 46).

Culture media from HIV-infected macrophages also contained large amounts of ADP, AMP and a low concentration of ADO. Notably, however, extracellular ATP binding its receptor P2X7 was the only agonist shown to trigger rapid release of virus containing compartment sequestered HIV-1 virions without simultaneously causing the death of infected macrophages (47). In contrast, extracellular ATP acted in a P2X7 receptor dependent manner to protectively restrict viral replication and facilitated interferon ß (IFN-ß) secretion with subsequent promotion of antiviral immunity (48). These examples highlight how the same purinergic pathway agonist, extracellular ATP, may be regarded as either a friend or foe of viral infectivity according to the viral species.

Different viruses may favor activation of different purinergic mediators, for example vesicular stomatitis virus (VSV) induced the release of UDP (49, 50), whereas infection with respiratory syncytial virus (RSV) caused predominantly UTP release in mice, that subsequently participated in signaling pathways that reduced basal alveolar fluid clearance (AFC) a symptom of lower respiratory tract disease (51). In RSV-induced goblet-cell metaplastic cultures and in bronchoalveolar lavage of influenza H1N1 infected mice, extracellular ATP and ADO accumulation was described (52). Release of these nucleotides and ADO may exacerbate lung inflammation and consequent tissue damage (53). Accordingly, acute injury of the lung tissue due to the viral-induced immune response was greatly reduced in A1 ADO receptor-knockout mice, highlighting a role for these receptors in lung pathogenesis due to influenza virus (54). ATP release was also found in VSV-infected RAW 264.7 and L929 cells (48) and in primary human bronchial epithelial cultures infected by RSV (55).

## Purinergic Cell Signaling can Impair Viral Function

**Figure 2:**
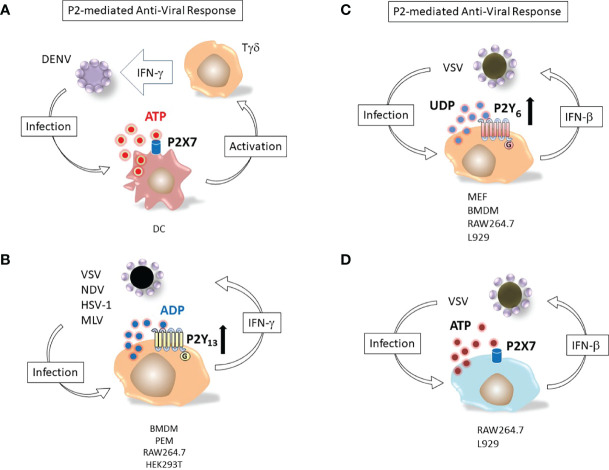
Nucleotide induced defensive responses. **(A)** ATP and P2X7 dependent immunological response to DENV. Dendritic cells (DC) and Tγδ lymphocytes (Tγδ) major responders against Dengue-2 virus (DENV) infection. Binding of DC P2X7 receptor by extracellular ATP activates Tγδ to produce of interferon-γ (IFN-γ), potentiating the antiviral immune response (59). **(B)** ADP and P2Y_13_-dependent antiviral response. Replication of viruses as different as vesicular stomatitis virus (VSV), Newcastle disease virus (NDV), herpes simplex virus 1 (HSV-1) and murine leukemia virus (MLV) is restricted by massive release of ADP from infected cells; (IFN-γ) up-regulates the ADP activated P2Y_13_ receptor (60). **(C)** UDP and P2Y_6_-mediated response against VSV infection. Murine embryonic fibroblasts (MEF), bone marrow-derived macrophages (BMDM), RAW264.7 or L929 cell lines release UDP upon infection with vesicular stomatitis virus (VSV). The nucleotide activates P2Y_6_ receptor and induces interferon-β (IFN-β) secretion (49). Bone marrow-derived macrophages (BMDM), peritoneal macrophages (PEM). **(D)** ATP and P2X7-mediated response against VSV infection. RAW264.7 or L929 cell lines release ATP upon infection with vesicular stomatitis virus (VSV). The nucleotide activates P2X7 receptor and induces interferon-β (IFN-β) secretion (48).

The multiple mechanisms underlying purinergic receptor participation in viral biology are attracting the attention of virologists involved in different fields (48, 56, 57). Human anti-viral response is based on transcription of genes encoding interferons, a family of cell host proteins endowed with the ability to potentiate the overall immune response thus heightening body anti-viral defenses (58) (**Figure 2**).

P2Y_13_, a receptor for extracellular ADP, was upregulated in mice treated with interferons (IFNs) and its expression protected mice from different viruses by inhibiting the cAMP/EPAC1 signaling pathway (60). P2Y_13_ mediated the antiviral response against vesicular stomatitis virus (VSV), Newcastle disease virus (NDV), herpes simplex virus 1 (HSV-1) and murine leukemia virus (MLV) while the other ADP-activated subtypes (P2Y_1_ and P2Y_12_) did not (60).

The interplay between nucleotides and IFNs in antiviral responses was also true for extracellular UDP. This uridine nucleotide was found to be released by infected cells and prompted IFN-β secretion by activating the antiviral response mediated by p38/JNK- and ATF-2/c-Jun-signaling pathways (49). VSV-infected cells showed an enhanced expression of the UDP receptor P2Y_6_. Consistent with the hypothesized protective role of this subtype, MRS2578, a selective P2Y_6_ antagonist that knocked-out expression of the receptor, increased VSV replication (49). Purinergic stimulation of P2 receptors during virus infection also modulated physiology of infected tissues. Instillation of the P2 antagonists suramin or XAMR-0721 in the airway of mice infected by the human Respiratory Syncytial Virus (hRSV) restored the alveolar fluid clearance capacity of the bronchoalveolar epithelium (61). Degradation of UTP but not ATP had the same effect, suggesting that a uridine activated nucleotide receptor (i.e. of the P2Y subtype) was likely involved in the hRSV-induced deficit of alveolar fluid clearance and that UTP was released into alveolar fluids as a consequence of hRSV infection (61).

A role for the P2X7 receptor subtype in controlling Dengue-2 virus infection in human monocytes has been shown (62). Dengue virus particles infect host cells by receptor-mediated endocytosis, and upon acidification of the endocytic vesicle, the nucelocapsid contents is released into the cytoplasm, where the viral genome is translated into a single polyprotein chain, subsequently subdivided into individual viral proteins by virus and host proteases (63). Pharmacological stimulation of human monocytes with extracellular ATP prior to Dengue-2 (DENV) virus treatment was able to inhibit infection, likely due to activation of P2X7-triggered antiviral responses, since specific inhibition of this subtype expressed in the host cell abrogated this effect (62). Stimulation of monocytes with ATP induced production of reactive oxygen species and nitric oxide, while it reduced secretion of CCL2, CXCL10 and chemokines IL-8 and TNF-α (62). Involvement of P2X7 in the human immune response to DENV infection was also shown. Accordingly, inhibition of the receptor expressed by dendritic cells (DC) significantly reduced the anti-DENV IFN-γ response of γδ T cells (59).

Over-activation of the P2X7 receptor has been linked to severe tissue damage due to an uncontrolled host immune response (64). The presence of viruses or even of adenoviral vectors in co-cultures of macrophages and epithelial cells induced P2X7-dependent inflammasome activation, causing acute respiratory distress syndrome and high mortality in P2X7-expressing mice but not in P2X7 KO mice. According to the well-known pro-inflammatory role of the receptor, the increased survival of P2X7 deficient mice was paralleled by decreased IL-1β, IL-6 levels and reduced neutrophil infiltration (64).Viral infection could induce over-expression of single P2 receptor subtypes. Peripheral blood mononuclear cells (PBMC) isolated from hepatitis C virus (HCV) patients responding to antiviral therapy (PEGylated interferon and Ribavirin) showed an increased expression of the P2X7 and P2X4 mRNA, while cells from non-responders had no up-regulation of messengers of these subtypes (65). Although the role of P2X7 and P2X4 subtypes in HCV infection is still not clear, authors have hypothesized that the latter might have a proviral role as they found increased expression of this subtype as a consequence of over-expression of E1/E2 HCV proteins in huh-7 cells (66). Changes in cell host P2 expression also occurred during Epstein-Barr Virus (EBV) induced cell transformation. In particular, both P2X7 mRNA and protein levels were potently augmented by the presence of EBV, while on the contrary, P2X1, P2X4, P2Y_1_ and P2Y_11_ proteins were down-regulated (67).

## Purinergic Signaling as a Viral Accomplice

The first scientific evidence that purinergic receptors were involved in the mechanism of virus entry in eukaryotic cells came from a report on hepatitis B virus (HBV) and hepatitis delta virus (HDV), both major causes of acute and chronic hepatitis (68). Generic inhibitors of the P2 receptors such as suramin, were found to block HDV entry into primary human hepatocytes. The same effects were observed for pyridoxal-phosphate-6-azophenyl-2’,4’-disulfonate (PPADS), a P2X antagonist and brilliant blue G (BBG), a selective inhibitor of the P2X7 subtype. It was thus suggested that this last receptor contributed both to viral penetration into hepatocytes and liver inflammation, i.e. hepatitis (68, 69).

Numerous observations concerning involvement of different P2Y and P2X receptors in human immunodeficiency virus 1 (HIV-1) infection of eukaryotic cells have concurred to give a more precise model of the interplay between cell receptors for HIV-1 binding/recognition, ATP-releasing molecules (connexins, pannexins, etc.) and purinergic receptors (19, 45, 46). HIV-1, infects the cell by binding CD4 (primary receptor) with viral glycoproteins gp120 and gp41 plus interactions with the chemokine receptors (secondary receptors or co-receptor) CXCR4 (expressed on lymphocytes) and CCR5 (present in macrophages and peripheral lymphocytes. It has recently been shown that P2 purinergic receptors play a fundamental role in allowing HIV-1 to penetrate eukaryotic cells and replicate, contributing to virion release and disease progression (45, 70–72). Hence, activation of different P2 subtypes (P2Y_2_, P2X1, P2X7) have been shown to modulate crucial steps of HIV-1 biology (44). Regarding questions about the mechanism adopted by the virus to evoke ATP release, it has emerged that upon virus-host-cell interaction there is opening of pannexin-1 (Panx1) membrane channels with consequent release of ATP and autocrine triggering of the P2Y_2_ receptor (45) with proline-rich tyrosine kinase 2 (Pyk2) kinase activation and fusion of Env-expressing viral membranes with membranes of the host cell. Accordingly, blocking just one of the pathway constituents (e.g. Panx-1, ATP, P2Y_2_ or Pyk2) caused a decrease in HIV-1 infection and replication efficiency. Another indication of the importance of extracellular ATP during HIV-1 infection came from the observation that the ATP hydrolyzing enzyme apyrase greatly reduced HIV-1 infection (45).

Another interesting point is that HIV-1 infected both CD4^+^ lymphocytes and macrophages although it was released through a cytopathic effect only by lymphocytes. In macrophages, the new progeny of HIV-1 virions accumulated into intracellular virus-containing compartments, but little was known about their release. It has recently been shown that ATP can stimulate the release of virions from infected macrophages through stimulation of the P2X7 subtype in the absence cell death (72). Tat HIV-1 exerts direct neurotoxic effects, but much less is known about indirect Tat-induced neurotoxicity and it has recently been reported that HIV Tat-mediated neuronal death can be caused by stimulation from ATP released by astrocytes (73). Hence, Tat upregulated P2X7 expression in astrocytes and its activation evoked intracellular calcium concentration increase, phosphorylation of ERK1/2, with subsequent secretion of CCL2 and ATP from astrocytes (73). Interestingly, P2X7 knockdown in astrocytes reduced Tat-mediated indirect neurotoxicity. Another cell type strongly involved in neuronal damage in HIV-infected individuals is the microglia, at least in primary neuron-glia co-cultures from mouse striatum. Although microglial cells express the P2X7 receptor, the P2X4 subtype has been indicated as a candidate for Tat-dependent neuronal injury (74).

T_reg_ lymphocytes play a fundamental role in limiting the immune response, promoting self-tolerance and preventing chronic inflammation and autoimmune diseases (75). However, their activities towards other lymphocytes prevent them from performing an efficient anti-viral response (76). The ectonucleotidase CD39 plays a role in down-modulating the immune response of T_reg_ lymphocytes. Low clinical outcome in HIV infected patients was associated with a high frequency of CD39^+^ T_reg_ cells suppressing *il-2* gene expression in CD4^+^ lymphocytes more efficiently than CD39^-^ T_reg_ cells. The CD39/ADO pathway activated A_2A_ receptor expressed by CD4^+^ lymphocytes thus increasing intracellular cAMP and reducing *il-2* gene promoter demethylation, with consequently reduced IL-2 production (77). T_reg_ inhibitory effects were counteracted by down-modulating CD39 expression while they were mimicked by P1 receptor agonists (78). Confirmation of the role played by CD39 and ADO in the pathogenesis of AIDS came from the observation that expansion of CD39^+^ expressing T_reg_ correlated with lower CD4^+^ counts in HIV-1 infected patients and more importantly, a *CD39* gene polymorphism associated with down-modulation of *CD39* expression resulted in slower progression to AIDS (78). Attention should be paid also to the interplay between thromboregulation and antiretroviral therapy, since cardiovascular diseases increased in patients subjected to highly active antiretroviral therapy (HAART) (79). Increased platelet aggregation was found in HAART treated patients compared to a control group. Since nucleotides play a role in blood coagulation and nucleotidases (E-NTPDase, E-5’-nucleotidase) were expressed on the platelet surface, the authors assessed their activity. E-NTPDase mediated ATP hydrolysis was higher in HIV infected individuals compared with uninfected controls and the HIV/HAART patients. Alternatively, ADP hydrolysis by E-NTPDase was higher in HIV patients compared to controls, and lower in HIV/HAART patients compared to controls and HIV infected individuals (80)

## SARS-CoV-2 Interactions With the Purinome

Much about the mechanisms of SARS-CoV-2 entry into the target cell determines the host and tissue-specific tropism, pathogenicity and zoonotic transmission (81). Prominently the viral spike protein (S) mediates fusion of the viral envelope and host cell membranes through its binding angiotensin-converting enzyme 2 (ACE2) (82), to date the only *bona fide* primary receptor for SARS-CoV-2, the highly related SARS-CoV and more distant HCoV-NL63 viruses. Viral entry occurs through the interaction of the Spike (S) protein with the cellular ACE2 receptor (83), facilitated in many cases by the processing of the S protein by proteases, in particular furin, the transmembrane serine protease 2 (TMPRSS2) and endosomal cathepsins. Coronavirus entry cofactors e.g. Neuropilin-1 (NRP-1) and Cluster of Differentiation 147 (CD147) can interact with furin cleaved SARS-CoV-2 spike protein since furin preactivation of the S protein increases the binding affinity to its receptor and co-factors, resulting in a widespread infectious cycle introducing differences in comparison to SARS-CoV despite both viruses targeting ACE2 (84). With reference to other RNA viruses, HIV-1 infection was found to induce the opening of ubiquitously expressed Pannexin-1 (Panx-1) channels on the surface of peripheral blood mononuclear cells, leading to a release of ATP that could stimulate purinergic signaling pathways that enhanced viral binding, accelerating HIV infectious entry and contributed to the compromised synaptic function found in cases of HIV-associated neurocognitive disorder (85). It was proposed SARS-CoV-2 may also induce opening of the large ionic Panx-1 channels, allowing passive outflux of ATP, metabolites and other small anions likely to have a role in exacerbating the inflammatory response (86). Independent research has confirmed this supposition, moreover in contrast to the common cold coronavirus 229E, the SARS-CoV-2 S protein caused more sustained Panx-1 channel opening, resulting in relatively enhanced ATP, PGE_2_ and IL-1ß release. The S protein induced Panx-1 channel opening required ACE2, endocytosis and furin activity (87). Notably, Tenofovir an AMP nucleotide analogue antiviral used in the treatment of hepatitis B and HIV, inhibited Panx-1 mediated ATP release in a human liver HepG2 cell line, diminishing adenosine levels in the extracellular space (88), but whether this relatively inexpensive and widely-available drug could be repurposed for SARS-CoV-2 infection awaits further *in vitro* confirmation and subsequent clinical trials (89).

The assumption that insights from successfully treating influenza or HIV-1 with purinome-directed drugs might have direct relevance against SARS-CoV-2 was met with abrupt disappointment. Nucleotide analogues that could be effective antiviral drugs to disrupt the replication of several viruses did not necessarily work against the SARS-CoV-2 coronavirus polymerase (90). This was surprising given the relatively large 30,000 base SARS-CoV-2 genome size (about three times the size of the influenza virus genome), that should have rendered it susceptible to a drug targeting replication machinery, to induce many errors and mutations causing error catastrophe (91). However, unlike other RNA viruses, coronaviruses tend to mutate less because of a correction system (92) that includes a non-structural protein (nsp14-ExoN) encoding a proofreading exonuclease removing mismatched nucleotides during replication and transcription (93, 94). Extending pathological effects, nsp14-ExoN has also been shown to interact with host Inosine-5’-monophosphate dehydrogenase 2 (IMPDH2) protein, known to regulate NFκB activation and signalling leading to induction of cytokines IL-6 and IL-8, both proinflammatory mediators (95). The porcine epidemic diarrhea virus (PEDV) has also been found to express nsp14-ExoN and a crucial mutation lead to high genetic instability during viral replication (96). Small molecular inhibitors of the nsp14 exoribonuclease have become an attractive target for novel SARS-CoV-2 replication inhibitors (97, 98). Notably the Omicron variant had many more mutations than previous SARS-CoV-2 variants with most of the mutations in the spike protein and high conservation in 25 other proteins, including those for replication. The Omicron variant does have a mutation leading to a I42V substitution in the Nsp14 protein, yet this is distant from the active site and needn’t necessarily explain the significant increase in Omicron lineage mutations (99). Whether a greater mutation incidence indicates SARS-CoV-2 Omicron variants are more susceptible to any intracellular purinome-directed drugs has yet to be determined.

SARS-CoV-2 has shown greater similarity with other viruses with regard to a need for host HSP90 protein, a non-conventional class purine utilizing enzyme, as a chaperone for viral proteins, helping prevent proteasomal degradation. Targeting HSP90 could inhibit replication of Kaposi sarcoma-associated herpesvirus (KHSV) (100) hepatitis C virus (HCV) (101) and also the human coronaviruses MERS-CoV and SARS-CoV (102). In a similar manner, *ex vivo* experiments involving transcriptomic profiling of SARS-CoV-2 infected human cell lines have shown that HSP90 inhibition impaired viral replication and activation of pro-inflammatory genes in primary human airway epithelial cells (103).

Fatty acid synthase (FASN), a multifunctional cytosolic enzyme of 272kDa, uses NADPH to condense acetyl-CoA and malonyl CoA into palmitate. The late stages of HIV-1 replication are modulated by FASN and specific inhibitors e.g. Fasnall could reduce the number of infectious HIV-1 particles, as was also the case for other enveloped viruses (104). Human embryo kidney HEK293T cells engineered to downregulate expression of fatty acid synthesis pathway enzymes FASN or ACACA failed to support SARS-CoV-2 replication (105), consistent with a palmitoylation modification of the SARS-CoV-2 spike protein being essential for viral infectivity (106). In addition to FASN inhibition, targeting fatty acid metabolism with inhibitors to ATP-binding Vacuolar protein sorting 34 (VPS34), a member of the phosphatidylinositol-3-kinase lipid kinase family that controls the canonical autophagy pathway and vesicular trafficking, also inhibited SARS-CoV-2 replication (107). Orlistat a FASN inhibitor used in clinical trials against numerous viruses, may suppress SARS-CoV-2 replication by lowering the lipid synthesis required in replication organelles with prevention of viral palmitoylation (108). This would likely have particular applicability for post-entry inhibition during the early phase of SARS-CoV-2 infection. In patients experiencing a later pathological post-acute phase of SARS-CoV-2 infection, the emphasis of coronavirus disease shifted from viral interactions to host alveolar epithelial and endothelial injury from proinflammatory responses (109), threatening the possibility of acute respiratory distress syndrome characterized by pulmonary edema and respiratory failure with a mortality rate risk of 30-40%. The purinome has important roles to play in inflammation, tissue adaptation to low oxygen conditions and the hypoxia-inducible factors crucial in mediating the crosstalk between hypoxia and inflammation. The mammalian brain is highly oxygen dependent and numerous adaptive responses have evolved to protect brain O_2_ levels, among them an important homeostatic control whereby C1 noradrenergic neurons, that are powerfully excited by hypoxia, control heart rate and blood pressure. Evidence from rodents points to astrocytes in the pre-Bötzinger Complex, a critical site for generating breathing rhythm, responding to hypoxia with release of ATP that acts *via* P2Y_1_ receptors to excite inspiratory neurons and increase ventilation (110). Extracellular ATP is also rapidly broken down by ectonucleotides (e.g. CD39 and CD73) into extracellular adenosine diphosphate (ADP), adenosine monophosphate (AMP) and ultimately extracellular ADO that signals *via* the 4 types of P1 receptor. Together, P2 and P1 receptor signaling and ectonucleotidase activity shapes the hypoxic ventilatory response. How this network is governed and how alteration of its dynamics influence hypoxia and ATP release from injury of damaged/ruptured cells are precise details that have yet to be determined before one can contemplate ATP/ADO signaling-directed therapy to stimulate ventilation (111).

Notably, ADO could stimulate expression of stress and inflammation related genes in folliculostellate cells including thrombomodulin and endothelial protein C (EPCR) that could play a negative-feedback role in controlling and limiting inflammation (112). Yet lung autopsies of COVID-19 casualties revealed a significantly low endothelial cell expression of the thrombomodulin and EPCR anticoagulants and this was associated with increased immune cell infiltration. Lack of SARS-CoV-2 particles in the lung endothelium implied that the dysfunctional endothelium and severe coagulation abnormalities might reflect pathological host responses in COVID-19 lungs rather than a direct infection with SARS-CoV-2 (113).

An observation consistent with the above interpretation was that like other viruses, SARS-CoV-2 induced a primary stress response in infected cells that evoked cellular senescence (114). This irreversible growth arrest of the cell was accompanied by a senescence-associated secretory phenotype (SASP) that could induce secondary senescence of endothelial cells, with an associated infiltration of macrophages and neutrophils resulting in endothelial damage and lung tissue thrombosis (115). Given involvement of the inflammasome in paracrine senescence (116) and purinergic P2Y_14_ receptor modulation of stress-induced hematopoietic progenitor cell senescence (117, 118), purinergic pathways may present new pharmacological avenues for controlling senescence-mediated morbidities from SARS-CoV-2 infection.

## COVID-19 Interactions With the Purine Signalling Pathway

Coronavirus disease 2019 (COVID-19) caused by SARS-CoV-2 infection has clinical features that vary widely between individuals from asymptomatic or mild symptoms to severe disease invoking pneumonia, acute respiratory distress syndrome, multiorgan failure and death. The pathobiology following SARS-CoV-2 infection involves several phases of COVID-19 evolution, typically an upper and then lower respiratory involvement, followed by a systemic inflammatory phase that may lack signs of SARS-CoV-2 presence, yet become excessive and cause cell death and tissue damage. Untargeted metabolomics analyses by liquid chromatography quadrupole time-of-flight mass spectrometry (LC/Q-TOF/MS) demonstrated that in contrast to healthy controls, COVID-19 patients bore significant differences in terms of purine, glutamine, leukotriene D4 (LTD4) and glutathione metabolism with evidence for an association between the pathophysiology of COVID-19 and altered purine metabolism (119). Concepts regarding SARS-CoV-2 co-involvement of the purine signalling pathway have been associated with three main phases from virus transmission to systemic hyperinflammation. In an early infection phase within the first 5 days of contracting the virus mild symptoms (fever, cough, fatigue, myalgia, sore throat, loss of sense of smell and headache) may emerge. In the second pulmonary phase, typically after 5-10 days, there can be respiratory failure (shortness of breath, hypoxemia, tachypnea) if this progresses, the third phase can include a hyperinflammatory reaction, acute respiratory distress syndrome, shock and multiorgan failure (120).

**Figure 3:**
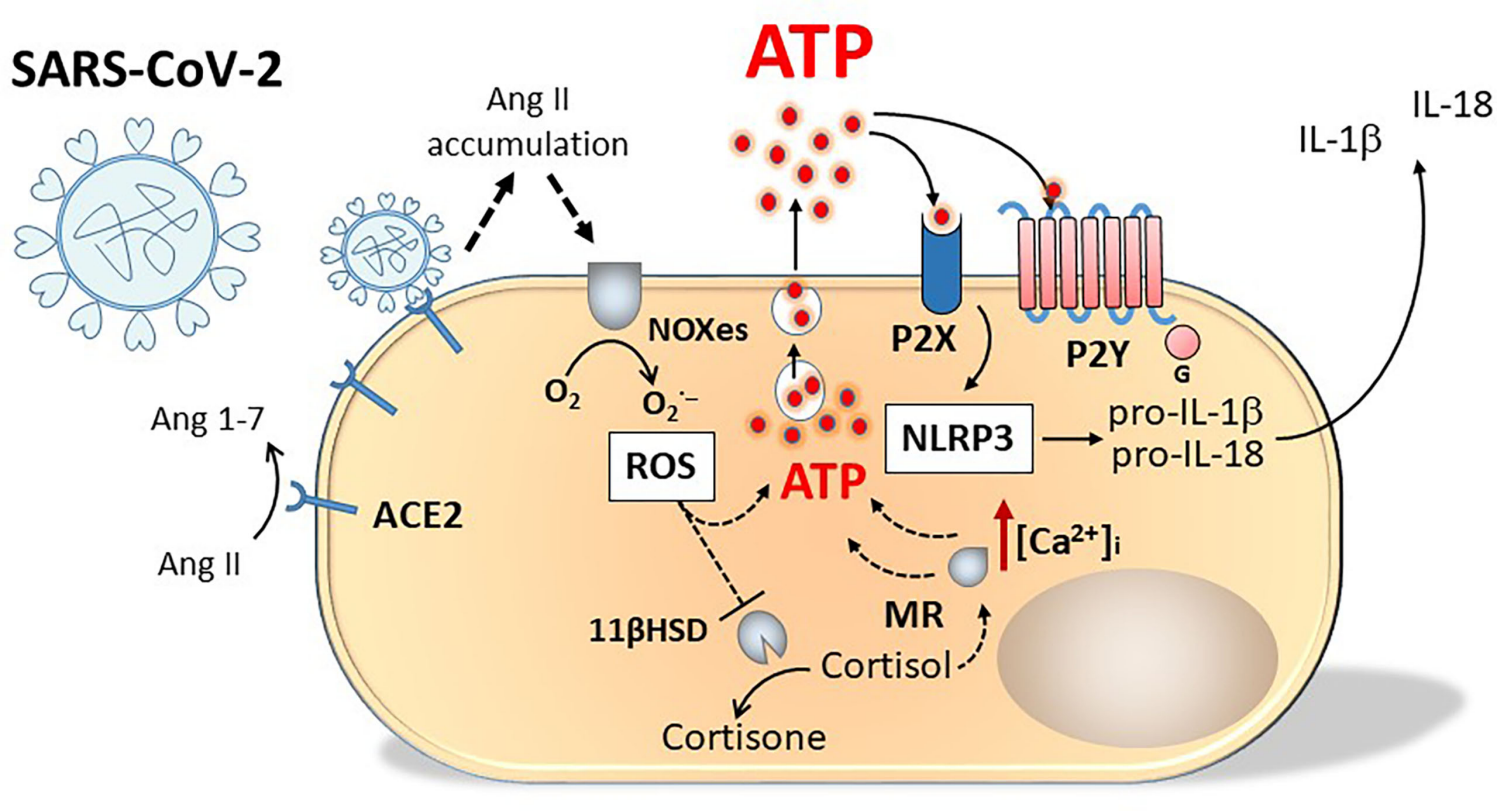
Schematic representation of purinergic signalling involvement in SARS-Cov-2 infection. Engagement of SARS-Cov-2 virus with ACE2 receptors causes angiotensin II accumulation and production of oxygen radicals (ROS) by NADPH oxidases (NOXes). ROS inhibit 11 beta-hydroxysteroid dehydrogenase (11ßHSD), the enzyme transforming cortisol into cortisone. Cortisol accumulation activates unprotected mineralocorticoid (type I) receptor (MR). Both ROS and MR stimulate ATP release from the cell. Activation of P2X and P2Y receptors by ATP increases intracellular Ca^2+^ concentration [Ca^2+^]_i_ evoking cell contraction and exocytosis of lysosomes containing ATP from the apical surface of the cell, resulting in a further increase in extracellular ATP concentration and in NALP-3 activation.

Asymptomatic or presymptomatic incubation periods for people testing positive for SARS-CoV-2 have facilitated pandemic spread. As a possible presenting symptom, about 20% of patients have reported loss of smell or taste impairment, with higher prevalence of such anosmia accompanying upper respiratory tract infections. Other viruses including the common cold virus and many rhinoviruses responsible for upper respiratory tract infections can also evoke post-viral anosmia that persists until damaged cells are repaired. The SARS-CoV-2 induced incidence of anosmia and also loss of taste or ageusia is high, without the virus directly invading the olfactory nerves and taste buds. The cells of these tissues lack the primary ACE2 receptor, nevertheless, the virus can target adjacent tissues e.g. ACE2^+^ nasal sustentacular cells or cells of the human salivary gland. By binding ACE2, the virus prevents the enzyme from converting angiotensin II to angiotensin, causing high angiotensin II levels to accumulate and stimulate NADPH oxidases (NOXes), leading to high levels of Reactive Oxygen Species (ROS) and a cascade of events leading to lysosomal release of ATP by damaged cells (121). The oxidative damage produced by ROS has numerous consequences, including perturbation of the corticosteroid type I mineralocorticoid (aldosterone) receptor (MR) and the enzyme 11ß-hydroxysteroid dehydrogenase (11ßHSD) a cortisol inactivating enzyme, that converts glucocorticoids (cortisol and corticosterone to cortisone and dehydrocorticosterone respectively) preventing them from binding the MR (122). Without functional 11ßHSD, cortisol could persist to activate the unprotected MR, stimulating release of ATP into the basolateral compartment of cells, that can act on purinergic receptors to open Ca^2+^ channels. The increased intracellular calcium can evoke contraction of the cell and exocytosis of lysosomes containing ATP from the apical surface of the cell resulting in further increased levels of extracellular ATP (123) (**Figure 3**). Acting as a neuromodulatory substance, ATP activated both P2Y and P2X receptors to markedly reduce the odour responsiveness of olfactory epithelium (124).

The highest levels of ACE2 expression have been found in the nose, with decreasing expression through the lower respiratory tract, a likely explanation for a gradient of SARS-Cov-2 infection that was higher in proximal versus distal pulmonary epithelial cells (125). This agreed with the concept of early infection in the upper respiratory tract (0-5 days) followed by aspiration and infection of the lower lung, whereby there can emerge a more severe pulmonary phase of disease in about 20% of cases according to WHO data. For most cases of non-critically ill classified patients, there was no difference in pulmonary function tests before and after SARS-CoV-2 infection (126). Early use of engineered ACE2 decoys may thus prophylactically mitigate lung disease (127). In contrast, for patients that succumbed to post-acute COVID-19 syndrome (PACS) where breathlessness persists, preliminary indications from novel xenon gas scan Magnetic Resonance Imaging (MRI) methods revealed there can be covert persistent lung damage that significantly impaired gas transfer to the bloodstream (128). Nuclear medicine methods are well suited to studying hyperinflammatory reactions and there are well-established radiopharmaceuticals targeting the purinergic P2X7 receptor (129). Measurement of Immunoglobulin (Ig) signatures based on IgM and IgG3 may provide early indication of a patient’s susceptibility to develop PACS (130).

Regarding acute cases of COVID-19, an induced pluripotent stem cell model of human airway epithelial lineage types indicated that multicilliated cells were primary targets of SARS-Cov-2 infection whereupon they could instigate interferon (IFN) and strong NFκB inflammatory responses, the hallmark of SARS-CoV-2 infection associated with morbidity (131). An initial release of cytokines and recruitment of immune reaction cells, e.g. CD4^+^ lymphocytes may progress excessively to trigger a so-called “cytokine storm” (132), causing progression from a difficulty in breathing to severe pneumonia, hypoxia, organ failure and risk of death.

Beyond susceptibility to viral infection, a second component for the propensity to develop a qualitatively different critical illness with distinct phenotypes to mild disease, involves activation of the innate immune system by virus infected cells causing macrophage and NK cell migration to the site of infection with release of pro-inflammatory cytokines. After viral replication, cells become lysed, releasing numerous molecules and damage signals including ATP and subsequently large amounts of ADO that in turn can activate purinergic signaling (133). The acute inflammatory response includes blood vessel dilation, increased permeability and leakage of fluid into the alveoli characteristic of pneumonia, that may reflect elevated bradykinin levels (134). This can attract more CD4^+^ T lymphocytes, compounding further release of pro-inflammatory cytokines, generating a positive feedback hyperinflammatory response. Profiling bronchoalveolar fluid in a comparison between mild or critical COVID-19 patients revealed not only numeric differences in T-cells exhibiting good effector function, but also a discriminatory lower activity of anti-viral type 1 and II IFN signalling pathways in critical COVID-19 cases. Reflecting a responsiveness to IFN signals, macrophages exhibited a pro-homeostatic M2 (anti-inflammatory) phenotype (135) helping to resolve inflammation in mild cases of COVID-19, whereas samples from critical COVID-19 patients had more M1 (proinflammatory) macrophages demonstrating an ATP-purinergic signaling inflammasome and increased levels of ATP in the bronchoalveolar lavage (136).

Extracellular ATP and the purinergic system have a homeostatic role in lung tissue. Alveoli are stabilized during respiration by a reduction in surface tension mediated by a surfactant composed of lipids and specific proteins secreted from the lysosome-related lamellar body organelles of alveolar type II cells. In addition to being important for preventing respiratory distress syndrome, the surfactant contributes to the innate immune system of the lung. The lamellar body membrane expresses a range of P2 purine receptors including the P2X4 receptor (137) with a role in boosting surfactant secretion and fluid clearance during homeostatic responses to ATP controlling local blood flow and surfactant secretion (138). These mechanisms remain also responsive to danger signal rises in ATP levels, triggered by aggravated airflow, changes in mucus hydration and viral infection. Inflammatory pulmonary hypoxia will result in increased extracellular ATP, promptly metabolized into ADP and then AMP by NTPDase that is then hydrolyzed by CD73 to ADO. The effects of ATP, ADP and ADO on tissue remodeling and inflammatory processes can diverge from acute anti-inflammatory and protective effects to chronic pro-inflammatory damage. No specific treatments targeting potentiation of P2X4 receptor have yet been developed, but conceptually they could counteract danger signal rises in ATP by promoting P2X4 receptor mediated Na^+^ transport into the cell cytoplasm, removing osmotically active Na^+^ from the alveolar lumen, thereby protecting the alveolus by restoring surface tension.

Intuitively, one might imagine that patients with a pre-existing pulmonary disease may be automatically predisposed to more severe COVID-19 symptoms. However, worldwide data initially reported the phenomenon that cystic fibrosis (CF) patients had a relatively improved Covid-19 survival, even though the defect in cystic fibrosis transmembrane conductance regulator protein (CFTR), impairing chloride and bicarbonate secretion, causes impaired mucociliary clearance and an exaggerated proinflammatory response that could be driven by infection (139). Moreover, the muco-inflammatory environment of CF airways often showed relatively high IL-1ß expression, an inducer of ACE2 expression. This would conceivably allow increased SARS-CoV-2 infection of more distal alveolar epithelial cells. Nonetheless, prior studies for precedent SARS-CoV infection had associated increased ACE2 pathway expression with improved lung function, perhaps by counterbalancing Spike protein-mediated downregulation of ACE2 (140). It has been proposed that the survival benefit demonstrated by homozygous CFTR mutations may involve purinergic pathways, since CF patients may show increased mitochondrial ATP production and elevated systemic levels of ATP (141). However, precise mechanisms were conjectured and more recent multicentric studies have indicated that COVID-19 should not be considered a mild disease in CF patients (142–144).

Cardiovascular disease following SARS-CoV-2 infection may reflect direct or indirect damage as a result of viral infection. The main mechanisms being proposed are inflammatory processes and an exacerbated host immune response involving extensive release of proinflammatory cytokines and chemokines. Myocardial injury increases extracellular ATP and ADO levels that activate different purinergic receptors. Extracellular ATP signals can activate P2X4 on the membrane of mast cells, macrophages and neutrophils that then further promote the inflammatory condition with increases in ATP and further proliferation of immune cells. Activation of T cells by the P2X1 receptor, with signaling of the P2X7 receptors of immune cells and also P2Y_1_ and P2Y_2_ in macrophages, can also trigger further release of inflammatory mediators such as IL-1ß, GM-CSF, IL-6, IL-10, INF-γ, γ-chain cytokines, TNF-α and IL-17, thereby amplifying inflammation. Blockade of purinergic receptors and suppression of ATP may be a strategy that helps inhibit purinergic signaling at P2 receptors, with the aim of reducing systemically mediated inflammatory damage to myocardial tissue (145).

Patients progressing to severe COVID-19 pneumonia, can suffer hypoxemia from both inflammation-compromised alveolar function and endothelial dysfunction together with a concerning systemic cytokine storm response ultimately causing micro and macro thromboses in pulmonary vessels and other organs. Colossal platelet activation, following sustained tissue factor (TF) expression in pathologically affected tissues and released procoagulant microvesicles can cause severe coagulopathy (146). The extensive platelet activation in COVID-19 patients was unlikely to reflect a direct effect of the virus, given low SARS-CoV-2 titers in the blood of only a low percentage of infected patients. Rather, numerous host factors as mentioned above, including IL-6 were implicated. IL-6 did not directly activate platelets *in vitro*, but enhanced the effect of a cytokine storm by potentiating the effect of ADP or thromboxane. Notably, antagonists of platelet P2Y_12_ receptors, that bound ADP, inhibited thromboxane-dependent pathways of platelet activation to a marked extent, more significantly than aspirin (147). Blockade of the P2Y_12_ receptor could also partially perturb release of sIL-6R, the specific soluble receptor needed for IL-6 signaling *via* gp130 (148). Therapeutic approaches addressing platelet alterations during SARS-CoV-2 infection (148) have entered clinical trials with outcomes that may reflect how effectively the IL-6 pathway can be inhibited. The effect of P2Y_12_ inhibitors on non-critically ill COVID-19 patients did not increase odds of improvement and reduced hospitalization (149), whereas treatment with IL-6 receptor antagonists tocilizumab and sarilumab did improve survival outcomes among critically ill COVID-19 patients (150).

## Purinergic Targets for SARS-Cov-2 Induced Pathologies

Natural selection forces favoring evolution of SARS-CoV-2 variants with higher fitness and transmission rates generate variants of the virus with novel properties that may be diversly pathogenic, risking new public health vulnerabilities. With modulation of transmission advantage and immune evasion, five variants of concern (VOCs) have demonstrated these phenotypes to a varying degree, with rapid whole genome sequencing revolutionizing our insights from ongoing surveillance. Beyond the root A lineage defined by the Wuhan/WH04/2020 sequence, the Beta [B.1.351 in Pango nomenclature (151)] and to a lesser extent the Gamma (P.1) variants showed immune evasion *in vitro* and local geographical spread. More significant VOCs reached a global distribution causing significant waves of population infection with an increase in the effective reproduction number (R naught, the virus’s transmission among a population that has no immunity). A mutation at position 681 within the Spike protein polybasic furin cleavage site, changing to histidine for VOC Alpha (B.1.1.7) and arginine for VOC Delta (B.1.617.2), generated in each case variants that were 50% more transmissible than prior forms. In the latter Delta variant, this alteration caused enhanced fusogenicity and pathogenicity (152). This concerned how upon attaching to the cell surface and binding the ACE2 receptor, SARS-CoV-2 may exploit two different entry pathways to enter the cytosol (1): The early or cell surface pathway involving the virus directly fusing with the plasma membrane *via* help from the TMPRSS2 surface protease (2); The relatively late virus-triggered endocytic pathway, whereby virus is trafficked to the lysosome/late endosome where proteases such as cathepsin L prime the S protein for membrane fusion. SARS-CoV-2 Alpha, Beta and Delta variants displayed enhanced spike-mediated cell fusion with syncytia formation (153). Interestingly, P2X1-specific antagonistic compounds have implicated a role for this particular purinergic pathway in HIV-1 membrane fusion, although precise mechanisms remain uncertain (154).

Omicron, the fifth World Health Organization (WHO) nominated VOC, has emulated Alpha and Delta to achieve global dominance. The Omicron (B.1.1.529 lineage) first detected in Botswana, South Africa in mid-November 2021, has split into at least three divergent sublineages (BA.1, BA.2 and BA.3) of which BA.1 has spread rapidly around the world (155). The BA.1 Omicron genome encodes an unusual array of mutations, enigmatically evolved outside overt paths of transmission from previous variants, with at least 30 amino acid substitutions in the spike glycoprotein relative to the Wuhan/WH04/2020 sequence. Of these, 15 mutations were found in the receptor-binding domain (RBD) and within its receptor-binding motif (RBM) subdomain that interacts with the human ACE2 receptor, six out of nine mutations enhanced binding affinity. Overall, the Omicron RBD demonstrated over twice the ACE2 binding affinity of the original SARS-CoV-2 virus. However, whilst the Delta variant achieved efficient entry *via* fusion, Omicron has switched entry route preference, entering predominantly *via* the second endocytic pathway, reducing dependence on TMPRSS2-mediated activation. This offers a mechanistic explanation for significantly reduced syncytia formation by Omicron infected cells (156–158), yet also emphasizes how a selection pressure to evolve changes in fitness can make SARS-CoV-2 a moving target for treatments that seek to target early stages of virus-host interaction.

Focus on host pathological responses rather than viral infectivity *per se* is likely to be more useful for implementing therapies that target purinergic signalling involved in potentially more severe chronic aspects of COVID-19 disease (159) in particular post-acute sequelae of COVID-19 (PASC). Quantifiable risk factors for PASC are beginning to be resolved, four that have been determined include type 2 diabetes, RNAemia, Epstein-Barr virus (EBV) viremia and specific autoantibodies (160). The fact that one of the risk factors should be viraemia from EBV suggests that there may be commonalities among responses to virus that impinge upon the pathological potential. EBV reactivated from latency and entering lytic replication could induce ACE2 expression (161) or it may be that both viruses are implicated in compromising mitochondrial health (162). Notably, dissecting the individual roles of the 19 purinergic receptors encompassing the P2Y metabotropic and P2X ionotropic types has revealed close involvement in diabetes and metabolism (163), knockout mice engineered with an adipocyte-specific lack of P2Y_6_ receptor have a greatly improved glucose homeostasis with improved glucose tolerance and reduced systemic inflammation (164).

A number of different innate immune response cells form a first-line defense against viral infection; macrophages, neutrophils, platelets and mast cells having significant COVID-19 roles in activating the innate immune system that is critical for establishing a subsequent adaptive immune response mediated by numerous T lymphocytes. Principally, these include regulatory T cells (T_reg_) and T helper cells (Th), CD4-positive cells that release cytokines to “help” the activity of other immune cells. The interleukin twelve (IL-12)-induced Th1 subset generates interferon gamma (IFN-γ) and tumor necrosis factor (TNF-α) whereas the IL-6 driven Th17 cells generate IL-17 and IL-21 (165). Recent studies have shown significant reduction of T_regs_ in COVID-19 patients, with an imbalance in the Treg/Th17 ratio weakening inflammatory inhibition worsening patient prognosis (166).

The innate immune system is sensitive to pathogen-induced danger-associated molecular patterns (DAMPs) of expression, with responsive pattern-recognition receptors (PRR) including toll-like receptors (TLRs) (167), NOD-like receptors/nucleotide-binding domain and leucine-rich repeat containing receptors (NLRs) including NLR family pyrin domain containing 3 (NLRP3) (168) and retinoic acid inducible gene I (RIG-I)-like receptors (RLR) (169) that can sense viral RNA and collectively serve as components of an inflammasome that detects the pathogen’s presence with activation of an antiviral immune response, including production of type I interferon (170). Like other human respiratory RNA viruses, SARS-CoV-2 proteins can act to suppress innate immune responses (171, 172). The multifunctional nonstructural protein 5 (NSP5) encoded main protease (Mpro), has not only a pivotal role in the replication cycle of all coronaviruses, but acts to evade intracellular innate immunity (173) by targeting RIG-I and mitochondrial antiviral signaling (MAVS) proteins (174). There is evidence that the SARS-CoV-2 envelope (E) protein can have biphasic effects, initially suppressing the host NLRP3 mediated inflammasome activation during the early stages of viral infection helping to protect early viral replication. Yet in the presence of viral RNA derived ssRNA sequences, a non-classical pathway could activate the NLRP3 in human macrophages (175). Although E protein potentiation of this NLRP3 inflammasome activation should serve to advantageously limit viral replication and stimulate an adaptive immune response, it may aberrantly exacerbate inflammasome activation, inducing an autoinflammatory disease and tissue damage (176, 177). Hence, it has been hypothesized that overactivation of NLRP3 inflammasome by SARS-CoV-2 infection would lead to excessive production of master inflammatory cytokines such as IL-1β and IL-18 (178) and could also damage hematopoietic stem/progenitor cells (179).

A number of purinergic receptors are integral to a macrophage surveillance function sensitive to even slight changes in the tissue microenvironment. Of these, P2X7 receptors have a relatively low affinity to ATP, yet respond to high concentrations of extracellular ATP under pathological conditions and then again to still higher concentrations of ATP, when a threshold of repetitive agonist application causes P2X7 receptors to further interact with a range of other membrane channels (e.g. P2X4, transient receptor potential A1 (TRPA1), pannexin-1, ANO6 chloride channel), making membrane permeability increase further (180). In particular, the macrophage P2X7 receptor is coactivated with the lipopolysaccharide-sensitive toll-like receptor 4 (TLR4) to induce the formation of the inflammasome 3 (NLPR3) that then activates pro-interleukin 1ß (pro-IL-1ß) that can degrade caspase-1, leading to IL-1ß release. Concurrently, P2X7 receptor activation induced downstream upregulation of second messengers (e.g. phospholipase A_2_, p38 mitogen-activated kinase (p38MAPK) can activate reactive oxygen and nitrogen species and rho G proteins). Notably, elevated Rho GTPase and mTOR pathway activities were among features characterizing responses to SARS-CoV-2 that were distinct from those to HIV-1 infections (181). Data from corneal allograft rejection studies demonstrated that blockage of the ligand-gated ion channel P2X7 receptor suppressed Th1/Th17-mediated immune responses and NLRP3 inflammasome activation (182).

In addition, macrophage P2Y family receptors (135) consisting of seven hydrophobic transmembrane domains serve to bind nucleotides or nucleotide sugars at three extracellular loops whilst intracellular regions mediate G protein activation. Under stressful conditions, P2Y receptors also form homodimers and heterotrimers to diversify the biochemical spectrum of activities in response to released nucleotide ligands modulated by ectonucleotidases e.g. CD39 and CD73. COVID-19 patients have been shown to have relatively low CD73 levels on cytotoxic lymphocyte populations including CD8^+^T, natural killer T (NKT) and natural killer (NK) cells (183) and several such abnormal phenotypes may contribute to an uncoupling between the innate and adaptive immune system in patients experiencing progressive COVID-19 (184).

Neutrophils recruited by activated macrophages are the most abundant immune cells in the circulation. Prompted through TLR activation they release neutrophil extracellular traps (NETs) comprising a mesh of DNA-based cytoplasmic material containing antimicrobial elements such as granule proteins, cathepsins, neutrophil elastase and myeloperoxidase together with cytoskeletal proteins; a process finely regulated by purinergic signaling (185). Viruses e.g. respiratory syncytial virus (RSV) and influenza are known to trigger NET formation and with the requisites of neutrophil ACE2 receptor, TMPRSS2 activity and viral replication, SARS-CoV-2 has been shown to directly stimulate expressing neutrophils to release NETs (186–189) making inhibition of NET formation a potential therapeutic target for COVID-19 treatment (190, 191). At the same time neutrophils are actively involved in triggering the resolution phase of an inflammatory response. Activation of specific adenosine receptors can suppress NET formation *via* cyclic AMP dependent signaling and the FDA-approved drug Dipyrimadole can potentiate anti-inflammatory adenosine receptor signaling by inhibiting ectonucleoside reuptake and stabilizing intracellular cyclic AMP, with prevention of NET-dependent thrombosis. Additional antiviral and anti-oxidant activities may also contribute to promising initial clinical trials that suggest dipyrimadole may be of benefit for critically ill COVID-19 patients, warranting larger randomized controlled trials (192).

Platelets have important roles to play in the innate immune system and can directly contribute to NET generation when stimulated by viral activation, the interaction of platelets and neutrophils likely to be a relevant factor for thrombogenesis (193). Multiple mechanisms involving a global inflammatory reaction in COVID-19 patients can serve to activate platelets, be it diseased endothelium, increased viscosity or viral infection, considered plausible, despite debate regarding whether the platelet ACE2 expression demonstrated in murine platelets *in vitro* (194) is consistent for humans *in vivo* (195). Extrinsic coagulation pathways ultimately lead to the formation of thrombin, a proteinase that initiates platelet activation, aggregation and secretion of proinflammatory and signalling molecules including thromboxane A_2_. Ordinarily these responses provide controlled hemostasis, however SARS-CoV-2 infection was associated with alterations in platelets (148) resulting in enhanced inflammatory tissue factor (TF) expression and an atypical amount of platelet-leukocyte rather than platelet-platelet aggregates. This was not unique to SARS-CoV-2, since increased platelet-leukocyte interactions have been reported in critically ill patients succumbed to influenza A (H1N1) virus infections (196). Platelet P2Y_1_ receptor signaling *via* RhoGTPase was found to be necessary for platelet dependent leukocyte recruitment (197), moreover the study highlighted subtleties and complexities concerning purinergic signaling in platelets according to their contextual role. Platelet functional roles bifurcate between hemostasis (aggregation to stop bleeding) or host defense mechanisms against pathogens (platelet motility and induction of neutrophil chemotaxis). Different orthosteric site molecular docking interactions of P2Y_1_ receptor agonists, e.g. ADP or the P2Y_1_ specific agonist MRS2365 could affect the selection of downstream signaling events; MRS2365 induced platelet chemotaxis more effectively than ADP. Modulation of P2Y_1_ receptor signalling may be distinct according to the context, whether the platelets are functioning for homeostasis or within an inflammatory microenvironment with accordingly biased responses.

*In vitro* evidence indicated that a P2Y_12_ inhibitors e.g. clopidogrel or prasugrel could enhance the effect of aspirin to inhibit platelet activation (198) including platelet activation induced by plasma from COVID-19 patients an activity attributed to P2Y_12_ inhibitors not only blocking ADP, but by also impairing IL-6-mediated platelet activation (199). The different P2Y_12_ inhibitors showed different clinical efficacy, trial data suggested that prasugrel and ticagrelor were more effective than clopidogrel for cardiac therapy (200). Taking into consideration unique characteristics, there may be preferences for use of a particular P2Y_12_ inhibitor depending on the circumstances, safety indications, contraindications, cost, efficacy and dose. Routes of administration may assist application of purinergic signalling targeted therapies. For example, when the half-maximal effective concentration of the P2X7 receptor antagonist lidocaine needed to block hyperinflammation was excessive, adverse reactions were minimized by selectively inhibiting P2X7 receptors of the immune cells of the lymphatic system, by inducing clonal expansion of T_regs_ in local lymph nodes that subsequently migrated to impart systemic anti-inflammatory activity, notably improving outcomes in six critically ill COVID-19 patients (201).

Mast cells (MC), present in lung mucosa have been commonly associated with diseases involving allergic reactions e.g. asthma, yet could also recognize viruses by diverse mechanisms, e.g. TLR3 detection of viral RNA or RIG-I recognition of uncapped viral RNA. Notably, MC also express ACE2 receptors and serine protease tryp2, qualifying a route to host SARS-CoV-2. There is growing evidence that mast cell activation may have an important role in COVID-19 hyperinflammation (202). SARS-CoV-2 activation has been shown to release histamine stored endogenously in MC granules, increasing release of the chemokines IL-8, IL-6. Alveolar macrophages bearing SARS-CoV-2 activated TLR could produce the pleiotropic inflammatory cytokine IL-1, that can stimulated MC IL-6 production and in combination these cytokines contributed to hyperinflammation (203). Purinergic signaling has been strongly implicated in mast cell degranulation. Extracellular ATP activated ionotropic P2X4 receptors and enhanced antigen-triggered degranulation potentiating a high affinity IgE receptor (FcεRI)-mediated tyrosine kinase signaling cascade. Among multiple P2Y receptor subtypes, the P2Y_11_ receptor was involved in degranulation enhancement *via* the PI3K/protein kinase B pathway (204). Studies in rhesus macaques, that presented authentic mild to moderate forms of COVID-19 (205), have shown that SARS-CoV-2 triggered mast cell degranulation induced acute alveolar epithelial inflammation and lung injury and that prophylactic administration of MC stabilizers that blocked degranulation could dampen the SARS-CoV-2 response and prevent lung injury (206). Mast cell activation has also been associated with the unclear aetiologically of long COVID or PASC (207) and the repurposing of drugs addressing mast cell activation syndrome may help patients undergoing PASC rehabilitation training (208).

Of concern, lingering neurological symptoms, including impaired information processing, memory and concentration (aka “COVID fog”) are likely to reflect neuroinflammatory mechanisms involving brain-resident macrophages or microglia that can be induced even after relatively mild COVID-19 symptoms (209). Such persistent neurological complications may also follow infection with Ebola, Zika and influenza A viruses, though it remains unclear whether the infectious virus needs to persist in the affected tissue or whether infection alters the tissue to evade an innate immune response, with altered immune processes persisting to invoke impaired function of mitochondria and microglia causing neuroinflammation. Given their strategic role, it is not surprising that viruses should target mitochondria, the key organelles that govern cellular homeostasis, metabolism, innate-immune signalling, aging, apoptosis and other signalling pathways. Either targeted directly by viral proteins or influenced by changes in the cellular microenvironment during viral pathogenesis, the mitochondria constitute a dynamic population of organelles that continuously elongate (by fusion), divide (by fission) and undergo a controlled turnover with elimination by the process of mitophagy (210). Several viruses modulate mitochondrial functions that act as a central hub for the innate immune system. Under excessive stress the mitochondrial inner membrane can become depolarized releasing mitochondrial unique components including mtDNA, cardiolipin, N-formyl-peptides and mitochondrial reactive oxygen species (mROS) that constitute mitochondria-derived DAMPs (mDAMPs). These can bind to and activate the NLRP3 inflammasome that is colocalized with mitochondria *via* mitochondrial outer membrane proteins; MAVS and mitofusion 2 (MFN2). Together with the involvement of other interconnected organelles, mitochondria activated the cyclic guanosine monophosphate synthetase (cGAS)-stimulator of interferon genes (STING) signaling pathway, leading to the production of host defense type I IFNs and pro-inflammatory cytokines (211). Therefore, beyond producing the ATP that was released and necessary for driving systemic inflammation (39), mitochondria interacted with P2X1, P2X4 and P2Y_11_ receptors to regulate T cell metabolism (212), migration (213) and activation (214); emphasizing the important role of purinergic signalling pathways in persistent neuroinflammation (215).

## Discussion

There is growing appreciation that targeting purinergic receptors may bring benefit to treat SARS-CoV-2 induced processes associated with the pathologies of COVID-19 (216–222). Accumulated clues to the puzzle of SARS-CoV-2 have suggested that when a virus can initiate platelet and neutrophil driven exacerbated macrophage inflammatory circuits, this can spell a pandemic. The molecules of the purinergic signalling pathway receptosome are directly implicated as key mediators for the interactions between cells involved in these principle pathological processes of concern (223–226). Despite available vaccines and emergency use antibodies (EUA), a pharmacological prevention and treatment therapy of COVID-19 is still urgently needed, especially for PACS sequelae. Observations regarding viral infections demonstrated that P2X1 receptor inhibitors e.g. NF279 or NF449 may block HIV-1 productive infection and associated inflammation processes in cultured cells (227), or that absence of the P2X7 receptor in mouse models showed a better outcome to influenza virus infection (228). Such outcomes have yet to be translated into treatment for viral comorbidities in the clinic. Nevertheless, drugs targeting purinergic signaling affected processes are being repurposed with some success against acute COVID-19. Tranilast, an old anti-allergy clinical drug is a direct NLRP3 inhibitor that has entered clinical trials for the treatment of COVID-19 patients (229). Ticagrelor, an orally administered selective reversible P2Y_12_ receptor antagonist, preventing P2Y_12_ and ADP mediated platelet activation and aggregation has been approved for prevention of severe cardiovascular events and may be suited for sepsis-induced coagulopathy in COVID-19 (230) especially since in silico analysis suggests ticagrelor also bound the main protease and spike proteins of SARS-CoV-2 (231). Preliminary trials of prehospital antiplatelet therapy in patients with COVID-19 was associated with modest yet significantly lower in-hospital mortality (232). A number of new compounds targeting P2X and P2Y receptors are currently under development. Of these, Cangrelor an anti-platelet nucleotide analogue drug, derived from ATP, capable of acting as a high-potency competitive analogue for most P2Y receptor subtypes (233), also scored highly in computer models seeking potential candidates to inhibit the desirable target Mpro (234). Similarly, Entecavir Hydrate, a guanosine analogue possessing anti-HBV activity was a P2X7 antagonist that could ameliorate platelet activation and thrombus formation (235). It has recently been identified among nucleoside and nucleotide analogues as a potential novel SARS-CoV-2 RNA-dependent RNA polymerase (RdRp) inhibitor (236). It is possible to envisage that agents modulating purinergic signaling may be advantageously capable of a dual role against both SARS-CoV-2 replication and hyperinflammatory COVID-19 symptoms. The complex context-dependent nature of purinergic signaling makes it a difficult yet potentially versatile therapeutic target, therefore, further pre-clinical and clinical studies are needed to confirm preliminary observations that support modulation of SARS-CoV-2 mediated responses by purinergic signaling. Intense global research seeking to limit and effectively control the SARS-CoV-2 pandemic has accelerated new technologies that can greatly improve our understanding of purinergic signaling and the enormous shared scientific progress already demonstrated by an ability to provide a vaccine defense provides cause for optimism concerning ever-improved adjunct pharmacological interventions.

## Author Contributions

All authors listed have made a substantial, direct, and intellectual contribution to the work and approved it for publication.

## Funding

Local funds of the University of Ferrara.

## Conflict of Interest

The authors declare that the research was conducted in the absence of any commercial or financial relationships that could be construed as a potential conflict of interest.

## Publisher’s Note

All claims expressed in this article are solely those of the authors and do not necessarily represent those of their affiliated organizations, or those of the publisher, the editors and the reviewers. Any product that may be evaluated in this article, or claim that may be made by its manufacturer, is not guaranteed or endorsed by the publisher.
